# A phylogenetic and trait‐based analysis of community assembly in a subtropical forest in central China

**DOI:** 10.1002/ece3.6465

**Published:** 2020-07-10

**Authors:** Jiaxin Zhang, Nathan G. Swenson, Jianming Liu, Mengting Liu, Xiujuan Qiao, Mingxi Jiang

**Affiliations:** ^1^ Key Laboratory of Aquatic Botany and Watershed Ecology Wuhan Botanical Garden Chinese Academy of Sciences Wuhan China; ^2^ University of Chinese Academy of Sciences Beijing China; ^3^ Department of Biology University of Maryland College Park MD USA; ^4^ Center of Conservation Biology Core Botanical Gardens Chinese Academy of Sciences Wuhan China

**Keywords:** BGDS forest dynamic plot, community assembly, functional traits, null models, phylogenetic structure

## Abstract

Despite several decades of study in community ecology, the relative importance of the ecological processes that determine species co‐occurrence across spatial scales remains uncertain. Some of this uncertainty may be reduced by studying the scale dependency of community assembly in the light of environmental variation. Phylogenetic information and functional trait information are often used to provide potentially valuable insights into the drivers of community assembly. Here, we combined phylogenetic and trait‐based tests to gain insights into community processes at four spatial scales in a large stem‐mapped subtropical forest dynamics plot in central China. We found that all of the six leaf economic traits measured in this study had weak, but significant, phylogenetic signal. Nonrandom phylogenetic and trait‐based patterns associated with topographic variables indicate that deterministic processes tend to dominate community assembly in this plot. Specifically, we found that, on average, co‐occurring species were more phylogenetically and functionally similar than expected throughout the plot at most spatial scales and assemblages of less similar than expected species could only be found on finer spatial scales. In sum, our results suggest that the trait‐based effects on community assembly change with spatial scale in a predictable manner and the association of these patterns with topographic variables, indicates the importance of deterministic processes in community assembly relatively to random processes.

## Graphical abstract

Trait‐based and phylogenetic tests of subtropical tree community assembly across scales.

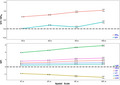

## 1 INTRODUCTION

Many evolutionary and ecological processes interact to shape species distributions in forests communities (Cadotte & Tucker, 2017; Kembel & Hubbell, 2006). Two types of niche‐based processes, environmental filtering and niche differentiation, are commonly invoked to explain the mechanisms of forest community assemblages (Chase & Leibold, 2003; Kunstler et al., 2012; Silvertown, 2004). Environmental filtering can be defined as the process of functionally similar species with superior performance in a given habitat (abiotic environment) excluding the presence of functionally dissimilar and inferior species (Keddy, 1992; Weiher, Clarke, & Keddy, 1998) or, more strictly, as the inability of a species to live in a given habitat irrespective of biotic interactions. (Kraft, Adler, et al., 2015). The first definition is now commonly referred to as competitive exclusion due to performance differences (e.g., Chesson, 2000), whereas the second definition can be referred to as environmental filtering sensu stricto. Along with these processes, niche differentiation or niche differences may preclude functionally similar species from co‐occurring due to competitive effects (Cahill, Kembel, Lamb, & Keddy, 2008; MacArthur & Levins, 1967). Larger niche differences promote species co‐occurrence, that is the more dissimilar two species are from one another, the more likely that they will be able to co‐occur, resulting in overdispersion of species co‐occurrence patterns as a result of competition or of enemy‐mediated density dependence (Barabás, Michalska‐Smith, & Allesina, 2016; Bennet, Lamb, Hall, Cardinal‐McTeague, & Cahill, 2013; Cavender‐Bares, Kozak, Fine, & Kembel, 2009; Chesson, 2013; Kunstler et al., 2016).

However, the relative importance of the processes in structuring forest communities is still not well understood. This uncertainty can be mitigated by using refined measures of how species utilized resources (i.e., their ecological roles) and an appreciation that multiple processes may be operating simultaneously within and across spatial scales (e.g., Shipley et al., 2012; Swenson & Enquist, 2009). Over the past two decades, ecologists have increasingly used two approaches for estimating the ecological roles of species for the purpose of inferring mechanisms of community assembly from observational data (Cadotte, et al., 2009; McGill, Enquist, Weiher, & Westoby, 2006; Swenson & Enquist, 2009; Webb, 2000; Xu et al., 2017). The first method draws on patterns of phylogenetic relatedness (Cavender‐Bares et al., 2009; Gerhold, Cahill, Winter, Bartish, & Prinzing, 2015; Ingram & Shurin, 2009; Pashirzad, Ejtehadi, Vaezi, & Shefferson, 2018), and the other one directly quantifies the functional similarity of species upon the basis of functional traits (Díaz et al., 1998; Kraft, Valencia, & Ackerly, 2008; Webb, Ackerly, McPeek, & Donoghue, 2002). In previous studies, ecologists suggested that if there were significant signals of phylogenetic conservatism in functional traits, the phylogenetic similarity could be treated as a proxy of functional traits to detect the ecological processes in a community (Revell et al., 2008; Swenson, Enquist, Thompson, & Zimmerman, 2007; Webb et al., 2002). In recent years, some studies have found that there was a mismatch between phylogenetic dispersion and functional dispersion, even when significant phylogenetic signals existed (Swenson & Enquist, 2009). Thus, phylogenetic relatedness alone cannot depict the functional mechanisms when species converge and diverge in different functional traits (Swenson, 2013; Swenson & Enquist, 2009) and may be very misleading without considering the patterns of trait diversity in the communities under study (Hao, Zhang, Zhao, & Gadow, 2018; Pavoine, Gasc, Bonsall, & Mason, 2013). However, it is impossible to measure all of the functional traits of species and, therefore, phylogenetic information may be used to indicate the presence of non‐random processes governing community structure with the understanding that even random phylogenetic community structure does not guarantee that only stochastic processes are at work (Swenson, 2019; Swenson & Enquist, 2009).

The possibility that multiple processes drive community assembly and that these processes may simultaneously operate within and across spatial scales is now accepted (e.g., Swenson & Enquist, 2009; Weiher et al., 1998). Given this, strong inferences of community assembly processes are best made through the analysis of community structure across scales (Messier et al., 2010). Thus, while measuring the phylogenetic and trait structure of communities is useful, the power of such analyses is only realized when quantifying phylogenetic and functional similarity across scales (Swenson et al. 2012; Paquette, Joly, & Messier, 2015; Whitfeld, Kress, Erickson, & Weiblen, 2012). In tree communities, spatial scale is often the most important determinant of the phylogenetic and functional structure of communities and, therefore, serves as a logical starting point for examining the scale dependency in community structure and assembly (Swenson, 2013).

Most of subtropical areas on Earth form an extremely arid desert or Mediterranean climate. However, due to the monsoon circulation and Tibetan Plateau, the subtropical region in China holds the largest evergreen broad‐leaved forest in the world (Song, 2004) and harbors abundant seed plants and endemic species (Ying, 2001). In this study, we present a phylogenetically trait‐informed analyses of tree community structure across four nested spatial scales in the Badagongshan forest dynamic plot (BDGS FDP), which is located at the northern edge of mid‐subtropical zone in central China to address the following questions: (a) whether the phylogenetic dispersion can reflect functional trait dispersion; (b) which processes are most likely dominating the community assembly of the forest; and (c) whether the results from the test are scale dependent and what the indicates regarding the potential processes driving community assembly.

## 2 MATERIALS AND METHODS

### 2.1 Study site

This study was conducted in a well‐protected typical old‐growth subtropical evergreen broadleaf forest in the Badagongshan (BDGS) National Nature Reserve (29°46.041′N, 110°5.248′ E), central China. The Reserve is located in the northern part of the Wuling Mountains with a monsoonal humid subtropical climate. The mean fog‐free days and rainy days per year are 220 and 170, respectively, and the mean annual precipitation is 2,105.4 mm. Mean air temperatures for the year, July and January are 11.5°C, 23.3°C, and 0.1°C, respectively.

During 2011, a 500 × 500 m plot was established in the reserve using total station following the same protocol of the Center for Tropical Forest Science (CTFS) long‐term forest dynamics plots (Condit, 1998). The plot was divided into 625 20 × 20 quadrats. All stems of freestanding shrubs and trees with diameter ≥ 1 cm at breast height (DBH, 1.3 m) were tagged, mapped, identified, and measured. In the first census, there were 186,575 live individuals of 232 woody species (139 deciduous and 93 evergreen), belonging to 53 families and 114 genera. Topographically, the 25 ha plot is characterized by flat ridges and steep slopes, with elevations ranging from 1,355 to 1,456 m. Hereafter, we will refer to the plot as the BDGS FDP.

A soil survey of the forest plot has been previously conducted in the BDGS FDP (Qiao et al., 2015) where maps, at the scale of 10 × 10 m, 20 × 20 m, 50 × 50 m, and 100 × 100 m, of 13 variables were produced. The variables are as followed: total carbon, total nitrogen, total phosphorus, carbon stable isotope composition (at 0–10 cm and 10–30 cm depth), soil pH, bulk density, soil temperature, carbon density at 0–10 cm soil depth, and nitrogen stable isotope composition in the subsoil layer. Because many of these variables co‐vary, we used a principle components analysis to reduce this redundancy and we used the PC scores for the first four axes for downstream analyses. Additionally, the elevation and convexity values for each 20 × 20 m plot have been recorded from the original survey of the plot and are used in downstream analyses in this work.

### 2.2 Measurements of functional traits

In this study, six leaf traits were measured: leaf area (LA), specific leaf area (SLA), leaf thickness (LT), leaf carbon concentration (LCC), leaf nitrogen concentration (LNC), and leaf phosphorus concentration (LPC). Variation in leaf area is related to a trade‐off between light capture and leaf temperature (Dolph & Ditcher, 1980; Swenson & Enquist, 2009); Specific leaf area (SLA) represents a trade‐off between construction costs and leaf life span (Wright et al., 2004); Leaf thickness is related to defense ability and tolerance to light intensity (Mendes, Gazarini, & Rodrigues, 2001); While leaf carbon, nitrogen, and phosphorus concentration are relevant to leaf structural composition, mass‐based maximum photosynthetic rate, and bioenergetics strategy, respectively (Cornelissen et al., 2003; Perez‐Harguindeguy et al., 2013; Wright et al., 2004).

Leaves were sampled from early June to mid‐September in 2012 and 2015. For each species, we selected 1–16 healthy adult individuals (there were 14 species with only 1 stem in the plot) and collected 10–40 intact fully expanded fresh leaves from each individual (Cornelissen et al., 2003). When sampling, we selected leaves exposed to the sun. For understory species, leaves were sampled from the top of the plants (Perez‐Harguindeguy et al., 2013). In total, trait data were collected from 910 individuals (mean DBH: 12.64 cm) from 162 species (98 deciduous and 64 evergreen species). These species accounted for 99% of the total basal area and frequency of stems in the plot. In the following, we describe the measurement protocol for each trait.

Leaf area (LA; cm^2^) was measured by scanning fresh leaves using a Canon CanoScan LiDE 110 portable electronic scanner (Canon Inc.), and the areas were calculated using ImageJ imaging software with petioles and rachis removed (Abràmoff, Magalhães, & Ram, 2004). Leaves from each individual were scanned, and the individual mean LA was calculated by dividing by the number of leaves of each individual. The species mean LA was the mean value of all the individual LA of that species.

Specific leaf area (SLA; cm^2^/g^−1^) was measured using the LA measurements and drying the leaves at 80°C for 48 hr where upon their dry mass was recorded. The SLA was calculated as LA divided by the dry mass. Each individual had one value of SLA. The species mean SLA was the mean value of all individuals within the species.

The leaf thickness (LT; mm) was measured at the center of the leaf lamina with electronic vernier calipers (Mitutoyo Co.), avoiding major leaf veins, that is, the thickness between the abaxial and adaxial surfaces of a fully unfolded developed fresh leaf (Seelig, Stoner, & Linden, 2012).

Mass‐based LCC (%), LNC (mg/g^−1^) and LPC (mg/g^−1^) were calculated from the oven‐dried leaves by first grinding the leaves using a ball mill (NM2000; Retsch, Haan, Germany). LCC and LNC were measured by Stable Isotope Mass Spectrometer (Delta V advantage, Germany), and LPC was determined following the molybdenum blue spectrophotometric procedure (M200 PRO Multiskan Spectrum Spectrophotometer, TECAN, Austria). Prior to analysis, all traits values were log‐transformed to correct for skewness. Species mean traits values were calculated to analyze the interspecies differentiation, ignoring the variations within species.

### 2.3 Phylogenetic community structure tests

A phylogeny representing the 162 species in the BDGS plot was constructed using the online informatics tool Phylomatic (http://phylodiversity.net/phylomatic/), which uses the APG III‐derived mega tree with reference to Flora of China (http://www.efloras.org). Phylogenetic branch lengths were estimated using the BLADJ algorithm in Phylocom 4.2 (Webb, Ackerly, & Kembel, 2008) with fossil ages and estimated molecular ages from Magallon and Castillo (2009).

Next, we calculated the phylogenetic signal in our trait data, which estimates the degree to which shared branch length is associated with trait similarity. We estimated phylogenetic signal using the K statistic and whether the observed K value significantly deviated from a distribution of random K values generated by randomizing trait data on the tips of the phylogeny 999 times (Blomberg, Garland, & Ives, 2003). *K* values < 1 indicate less phylogenetic signal in trait evolution on the phylogeny than expected under a Brownian trait evolution model, while *K* values of 1.0 indicate that the trait distribution on the phylogeny perfectly matches a Brownian motion expectation, and *K* values > 1 indicate a higher degree of trait similarity of related taxa than that expected from Brownian motion. Traits have significant phylogenetic signal if the observed *K* is greater than 95% of the null distribution. Calculations of the *K* statistic and randomizations were performed in the R package “phytools” (https://cran.r‐project.org/package=phytools).

Next, we divided the 25 ha plot into nonoverlapping quadrats of four nested spatial scales (10 × 10 m, 20 × 20 m, 50 × 50 m, and 100 × 100 m), we then calculated the mean nearest taxon index (NTI) for all quadrats at each spatial scale to represent the phylogenetic structure. Specifically, the NTI metrics is a standardized version of the mean nearest taxon distance (MNTD) for all species in each quadrat (Webb, 2000; Webb et al., 2002). The NTI was calculated with the following equation:

(1) $$\text{NTI}=-1\times {\text{(MNTD}}_{\text{obs}}\;-\text{mean}\left({\text{MNTD}}_{\text{null}}\right))/\text{sd}\left({\text{MNTD}}_{\text{null}}\right)$$

where MNTD is the mean of the phylogenetic distance for each taxa to its nearest relative within a local community, calculated for each quadrat; MNTD_obs_ represents the observed value of mean nearest taxon distance; mean (MNTD_null_) represents the mean value from a null distribution after taxa names are randomly shuffled 999 times; and sd (MNTD_null_) is the standard deviation of the null distribution. To determine whether the observed phylogenetic community structure differs from that expected by chance, we used a common null model to randomize phylogenetic relatedness among species by randomly shuffling 999 times the taxa names across the tips of the phylogeny (Swenson, 2014). This algorithm randomizes the relatedness of co‐occurring species to one another, while maintaining species richness weighting by each species' plot‐wide occurrence (i.e. the number of quadrats in which each species is found). During each of the 999 shuffles, null mean nearest taxon distance (mean MNTD_null_) between co‐occurring taxa were produced, constituting the null communities to which the observed value was compared.

Positive NTI values indicate a phylogenetically clustered structure of communities (i.e., co‐occuring species were more phylogenetic related than expected by chance), whereas negative NTI values suggest an overdispersed phylogenetically structure. At the whole‐plot level, the significant deviations of the mean NTI at each spatial scale from the null expectation were assessed by a two‐tailed Wilcoxon signed‐ranks test (see Kembel & Hubbell, 2006; Kraft & Ackerly, 2010; Kress et al., 2009). Phylogenetic analyses above were performed using the R package “picante” (Kembel et al., 2010). Lastly, across scales, we used a bivariate regression analysis to quantify the relationship between the NTI values and the four PC axis scores defining soil variation and elevation and convexity.

### 2.4 Trait‐based community structure tests

**FIGURE A1:**
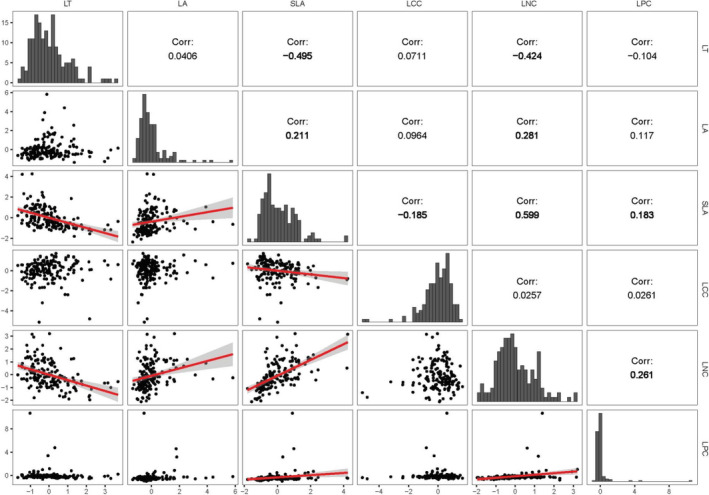
Frequency distribution histogram and Pearson's correlation coefficients for pairwise correlations between functional traits. Note. LT, leaf thickness; LA, leaf area (size); SLA, specific leaf area; LCC, LNC, and LPC, leaf carbon, nitrogen, and phosphorus concentration by mass, respectively. Boldface type indicates *p* < .05

Trait‐based analyses of community structure were also calculated at the four different spatial quadrate‐scales. The analyses were the same as the phylogenetic analyses with the exception that we used a functional trait dendrogram for each trait instead of a phylogeny. This helped facilitate a comparison between the phylogenetically and trait‐based tests. All analyses only included only species that had a value for a given trait. That is, if a species did not have a value for a given trait, it was not included in the observed or null model analyses. First, we calculated the nearest function index (NFI) to represent the functional dispersion in each quadrat, referring to the standard effect size of the nearest functional neighbor distance (NFND) from the functional dendrogram, while NFI_M_ referred to the nearest function index at multivariate trait space (calculated from the Euclidean distance matrix of six traits axes, and the Pearson correlation coefficients for pairwise correlations between trait was exhibited as Figure A1). The equation was as follows:

(2) $$\text{NFI}=-1\times \left({\text{NFND}}_{\text{obs}}-\text{mean}\left({\text{NFND}}_{\text{null}}\right)\right)/\text{sd}\left({\text{NFND}}_{\text{null}}\right)$$

A positive NFI indicates a functional clustered structure of communities, whereas negative NFI values suggest an overdispersed functional structure (Yang et al., 2014). The same null model was used in the functional trait analysis just like the analysis of phylogeny relatedness.

In addition, we also quantified the four moments of trait distributions: mean (along with variance), range, standard deviation, and kurtosis to interpret the functional composition of the study community, which are effective metrics for detecting deterministic community assembly processes (e.g., Kraft et al., 2008). The standard deviation of the nearest functional neighbor distance (henceforth SDNN), representing the regularity of the species spacing along a given trait axis (i.e., the standard deviation of the distance to the “nearest functional neighbor” in a community, irrespective of conspecifics in this calculation). Null trait distribution models were generated by creating 999 null communities of equal richness to the sample quadrat by drawing species randomly from the entire trait database after weighting by each species' plot‐wide occurrence. In addition, we calculated a plot‐wide abundance‐weighted null model, which maintained species richness and occurrence of each quadrat simultaneously. We predicted that, if environmental filtering was occurring at the quadrat scale, the variance of observed trait values and range were both reduced by the habitat‐based performance differences (i.e., smaller than the null values) (Cornwell, Schwilk, & Ackerly, 2006). Likewise, if niche differentiation were important, we predicted that the kurtosis would be smaller than expected by chance (community trait distributions with “fatter” tails and therefore may indicate an increase in the average trait disparity between co‐occurring species), while the SDNN would be lower (species spaced more evenly along trait axes), probably as a result of direct competition or negative density dependence (Kraft et al., 2008; Stubbs & Wilson, 2004; Wilson & Stubbs, 2012). The kurtosis and SDNN analyses were performed in the R package “fBasics” and “FD” respectively (Laliberte & Legendre, 2010). In the context of this paper, we viewed ecological null models as a proxy of neutral dispersal assembly process, since they led to random patterns of co‐occurring species (Gotelli & McGill, 2006; Hubbell 2005).

The significance of each metric was assessed using a plot‐wide Wilcoxon signed‐rank test by comparing the observed values of each quadrat relative to their respective null model expectation (Cornwell & Ackerly, 2009). Two‐tailed tests were applied to trait means, while one‐tailed tests were used for all other metrics in all analyses. As with the phylogenetic dispersion analyses, we used a bivariate regression analysis to quantify the relationship between the NFI values and the four PC axis scores defining soil variation and elevation and convexity.

Lastly, in order to determine whether functional and phylogenetic tests tended to identify the same quadrats had non‐random structure, we performed a chi‐square test to determine whether quadrats that had phylogenetic clustering or overdispersion also had trait‐based clustering or overdispesion at each spatial scale of analysis. All statistical analyses above were performed in the software R, version 3.1.3 (R Core Team, 2014).

## 3 RESULTS

### 3.1 Trait conservatism

All of the traits measured had moderate phylogenetic signal with K values ranging from 0.21 for LA to 0.37 for LNC. These weaker phylogenetic signals showed that all traits had less phylogenetic signal than the expectation of trait evolution under the Brownian motion model (all trait *K* values < 1; Table 1) and less variable than expected by random community phylogeny (all trait *p* values < .05; Table 1). SLA and LA had the least phylogenetic signal, while LCC and LPC had the most signal. In general, trait values from close relatives were more similar than expected by the null random model in the BDGS 25‐ha FDP.

**TABLE 1:** The ranges of functional traits and phylogenetic signal tests using Blomberg's K statistic

Trait	Range	*K*	*p*
LT (cm)	0.07–0.57	0.24	**.012**
LA (cm^2^)	1.93–341.98	0.21	**.032**
SLA (cm^2^ ** **g^1^)	21.76–761.74	0.22	**.02**
LCC (%)	19.08–61.65	0.34	**.001**
LNC (mg/g)	0.3–4.92	0.23	**.001**
LPC (mg/g)	0.05–1.69	0.38	**.001**

Note

Hereinafter: LT, leaf thickness; LA, species area (size); SLA, specific leaf area; LCC, LNC, LPC, leaf carbon, nitrogen, and phosphorus concentration by mass, respectively. Significant *p* values indicate that the phylogenetic signal differed from zero. *p* values < 0.05 are shown in bold.

### 3.2 Phylogenetic community structure

**FIGURE 1:**
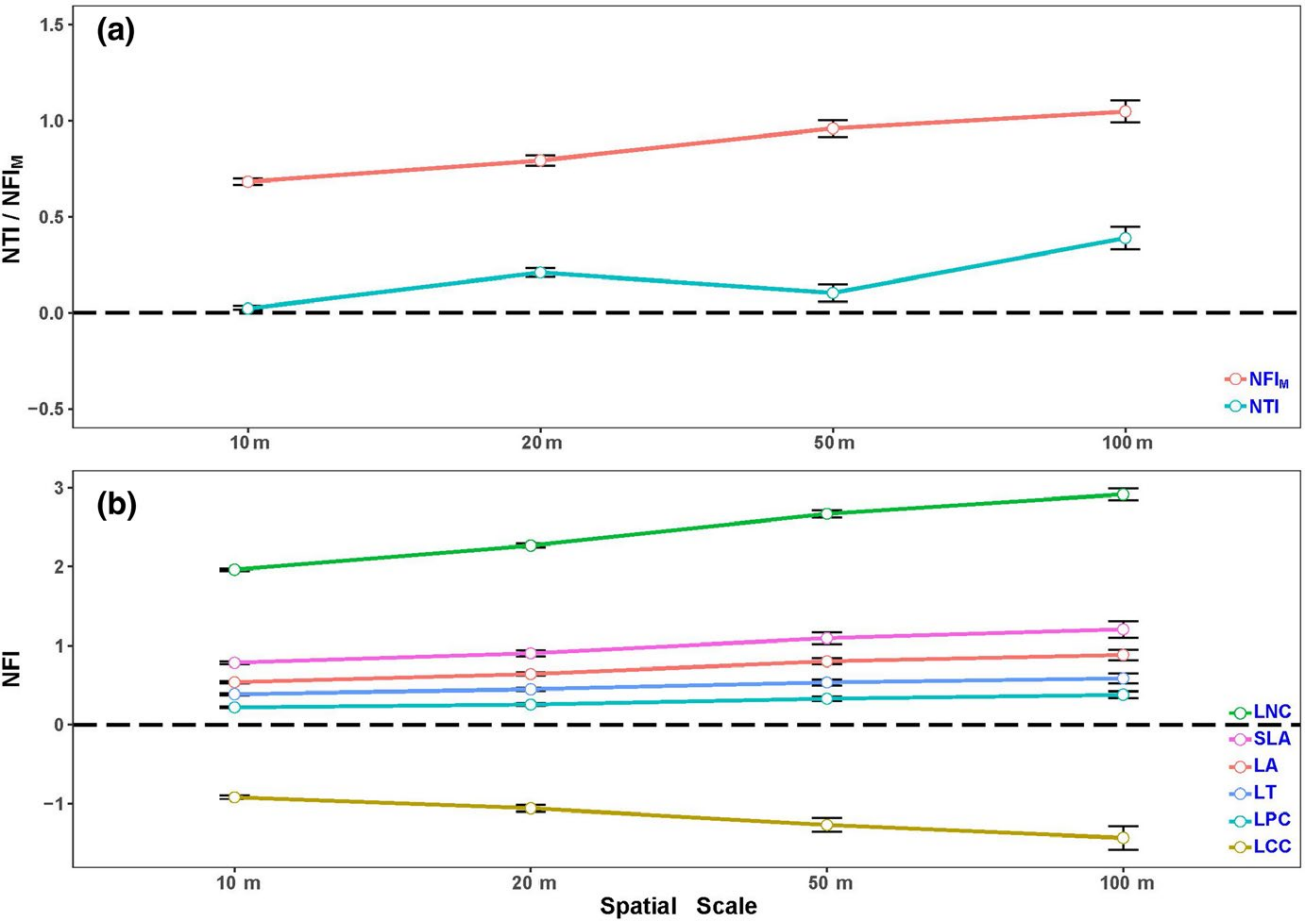
The pattern (Mean ± *SE*) of (a) nearest taxon index (NTI) and nearest function index of multivariate trait space (NFI_M_), and (b) nearest function index (NFI) of each univariate trait at four spatial scales within the 25‐ha BDGS FDP. Positive values indicate functional clustering, while negative values indicate functional overdispersion. LT, leaf thickness; LA, species area (size); SLA, specific leaf area; LCC, LNC, LPC, leaf carbon, nitrogen, and phosphorus concentration by mass, respectively

Compared with the more conservative occurrence‐weighted null model, the observed co‐occurring species tended to be more related than expected across spatial scales. Plot‐wide tests showed phylogenetic clustering at all spatial scales using the mean observed NTI metric (Table 2, Figure 1), though each spatial scale showed evidence of both clustering and overdispersion (quadrat‐level). However, at the smallest scale (10 × 10 m, 20 × 20 m), communities were not overdispersed for NTI.

**TABLE 2:** Results of a plot‐wide Wilcoxon signed‐ranks test determining phylogenetic and functional community structure at four nested spatial scales

Spatial scale	*N*	Mean richness	Nearest taxon index (NTI)					Nearest function index (NFI)				
			Estimated mean	*SE*	*p*	+^*^	−^*^	Estimated mean	*SE*	*p*	+^*^	−^*^
10 × 10 m	2,500	22.8	0.02	0.015	<.01	6.3	2.8	0.68	0.014	<.01	11.3	7.6
20 × 20 m	625	43.8	0.21	0.026	<.01	9.5	6	0.79	0.024	<.01	13.1	8.8
50 × 50 m	100	85.2	0.10	0.044	<.01	14	9.6	0.96	0.045	<.01	12	7.2
100 × 100 m	25	118.1	0.39	0.058	<.01	6	1.8	1.05	0.058	<.01	10	6.7

Note

Positive NTI/NFI values indicate phylogenetic and functional clustering, while negative values indicate phylogenetic and functional overdispersion of species occurring together in a quadrat. *N*: numbers of quadrats within the plot with corresponding mean richness listed. Significant *p* values indicate that the observed phylogenetic and functional structure at a given spatial scale differed from the null expectation, according to a plot‐wide Wilcoxon signed‐ranks test. +*: represents the percentage of quadrats exhibiting significant clustering while −*: significant overdispersion at *α* = 0.05.

Abbreviations: NFI, nearest function index; NTI, nearest taxon index; *SE*, standard error.

The NTI of subplots was related to elevation, convexity, and the first three PC axis (Tables A2 and A3). However, the relationship was not consistently significant across spatial scales. Specifically, soil PC axes tended to be related to NTI at the two largest spatial scales and variably related to NTI at the two smallest spatial scales. Elevation and convexity were both related to NTI at 20 × 20 m, but variably related to NTI at other scales.

### 3.3 Trait‐based assembly patterns

Unlike the phylogenetic analysis, which only detected clustering at all the scales, trait‐based analyses showed mixed signals throughout the BDGS FDP. The functional dispersion was significantly different from random for NFI and NFI_M_, and both of them were increased with spatial scale. NFI_M_ tests revealed functional clustering at all spatial scales of analysis. As for NFI tests, we found LT, LA, SLA, LNC, and LPC clustered across all spatial scales, while overdispersion occurred for LCC (Table 2, Figure 1).

The NFI was significantly related to elevation, convexity, and all soil PC axes across all spatial scales in this study (Table A2). However, at smaller scales, the direction and significance of the relationship between NFI and these variables was inconsistent.

**FIGURE 2:**
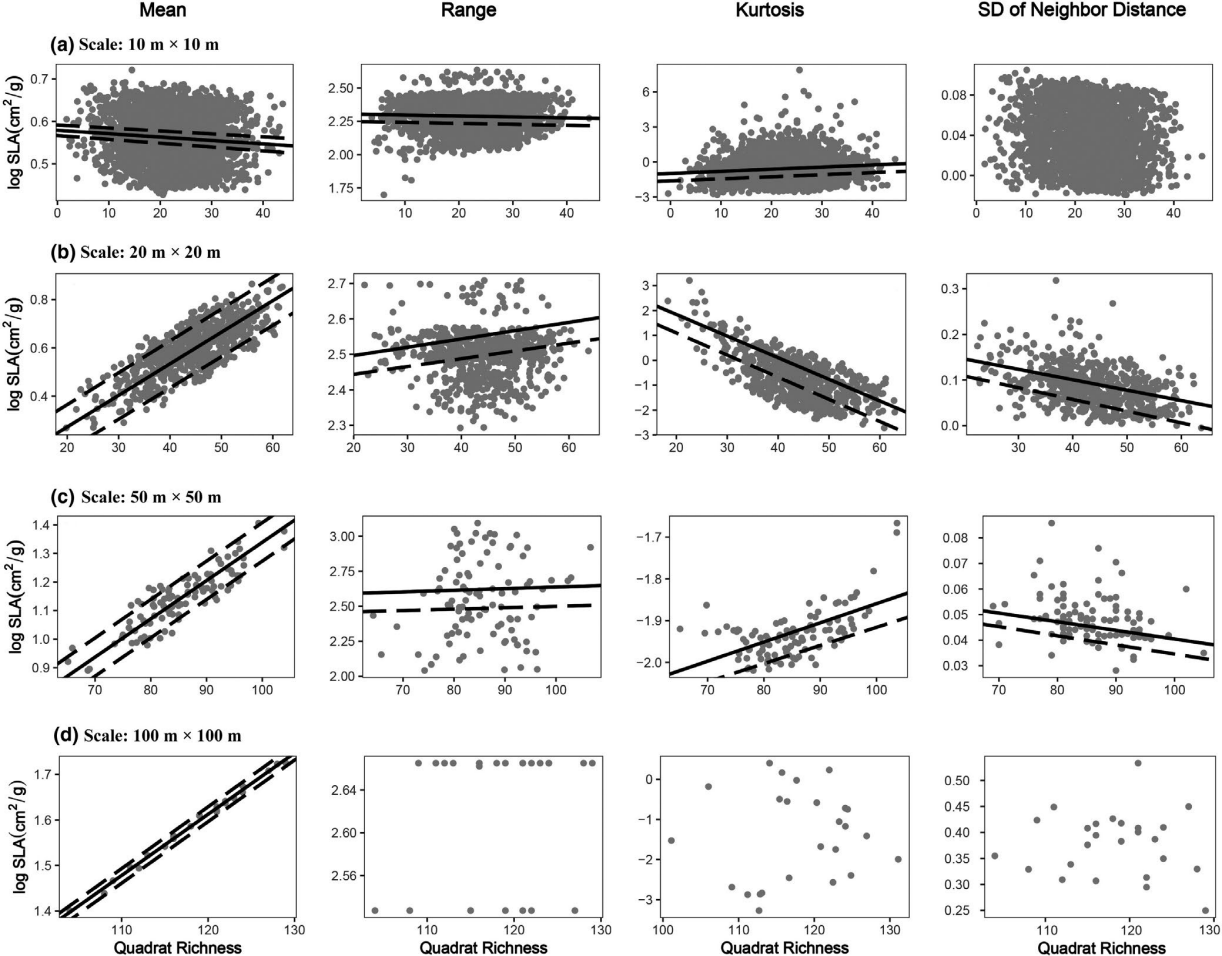
Examples of community trait distribution patterns at different scales. For instance, SLA (log‐transformed). Rows (a) (b) (c) (d) represented for mean, range, kurtosis, SDNN (standard deviation of nearest neighbor distance) of SLA at 10 × 10 m, 20 × 0 m, 50 × 50 m, 100 × 100 m scale, respectively. Points indicated the observed distribution of SLA in each quadrate as a function of quadrate richness. The solid line indicates the expected range value predicted by the null model, and the dashed line indicates the 5% confidence interval of the null distribution. One interval indicates one‐tailed test

On the one hand, trait variances and ranges were significantly smaller and markedly well‐separated within quadrats relative to the null expectation for most traits and most scales (Table 3, Figure 2), though mean standard effect sizes were small in many trait tests (Table 3). On the other hand, significant divergences with respect to kurtosis and SDNN relative to null expectation were found at the 10 m, 20 m, and 50 m scales, but not at the 100 m scale. LT and LNC represented significant clustering in all the four scales. However, on only one scale was overdispersion evident (50 m for LT and 20 m for LNC. Table 3, Table A1). For LA and LPC, there was significant clustering except on the largest scale, and significant divergences on the two small scales, but not the two large scales. For SLA, we can detect significant environmental filtering on all the four scales and significant niche differentiation except the largest scale. However, for LCC, significant environmental filtering was only found on 20 m scale.

**TABLE 3:** Result of plot‐wide trait‐based tests of community assembly with average effect sizes (±*SE*) at four nested spatial scales, (observed – expected)/null *SD*

Trait and Scale (m)	Environmental filtering			Niche difference	
	Mean	Range	Variance	Kurtosis	SDNN
LT					
10	0.77 ± 0.04	0.13 ± 0.02	**−0.115** ± **0.03**	0.54 ± 0.14	0.97 ± 0.01
20	1.132 ± 0.048	0.070 ± 0.05	**−0.089** ± **0.043**	0.099 ± 0.05	0.339 ± 0.038
50	**0.88** ± **0.02**	**0.213** ± **0.03**	**0.127** ± **0.07**	**0.23** ± **0.04**	0.53 ± 0.01
100	0.84 ± 0.049	**−0.042** ± **0.212**	0.617 ± 0.148	0.596 ± 0.017	0.992 ± 0.032
LA					
10	**0.87** ± **0.02**	0.28 ± 0.11	0.77 ± 0.03	−0.63 ± 0.04	**−0.14** ± **0.04**
20	1.181 ± 0.05	**−0.272** ± **0.038**	**−0.109** ± **0.057**	**−0.28** ± **0.05**	**−0.065** ± **0.05**
50	**0.97** ± **0.048**	**1.18** ± **0.05**	**0.94** ± **0.043**	−1.07 ± 0.05	−0.07 ± 0.038
100	1.013 ± 0.002	0.780 ± 0.052	1.699 ± 0.097	0.983 ± 0.007	0.030 ± 0.09
SLA					
10	**1.32** ± **0.03**	**−0.19** ± **0.06**	0.21 ± 0.03	**−0.19** ± **0.02**	0.33 ± 0.05
20	**2.314** ± **0.033**	−0.178 ± 0.05	**−0.208** ± **0.041**	**−0.17** ± **0.041**	**−0.167** ± **0.05**
50	**1.14** ± **0.06**	0.188 ± 0.03	0.334 ± 0.03	**−1.28** ± **0.014**	−0.93 ± 0.02
100	**0.992** ± **0.001**	0.787 ± 0.008	0.977 ± 0.149	1.203 ± 0.003	−1.019 ± 0.059
LCC					
10	0.77 ± 0.05	0.11 ± 0.04	0.45 ± 0.04	−0.17 ± 0.04	−0.81 ± 0.05
20	1.776 ± 0.058	**0.182** ± **0.033**	0.243 ± 0.048	0.055 ± 0.04	0.219 ± 0.058
50	0.47 ± 0.027	−0.39 ± 0.039	0.26 ± 0.043	−1.14 ± 0.099	−0.81 ± 0.088
100	0.462 ± 0.002	−0.445 ± 0.002	0.901 ± 0.127	0.214 ± 0.002	−0.883 ± 0.033
LNC					
10	0.33 ± 0.04	−0.88 ± 0.05	**−0.77** ± **0.02**	0.68 ± 0.11	0.56 ± 0.03
20	**1.667** ± **0.061**	−0.233 ± 0.05	**−0.032** ± **0.05**	**−0.22** ± **0.055**	−0.88 ± 0.042
50	0.63 ± 0.018	**−1.36** ± **0.049**	−1.39 ± 0.067	2.11 ± 0.031	1.15 ± 0.052
100	0.620 ± 0.001	−1.362 ± 0.001	**−0.774** ± **0.022**	−1.391 ± 0.001	1.155 ± 0.075
LPC					
10	0.87 ± 0.07	**−0.21** ± **0.14**	−0.33 ± 0.11	**−0.23** ± **0.06**	0.45 ± 0.03
20	1.270 ± 0.052	**−0.118** ± **0.044**	**−0.143** ± **0.045**	−0.108 ± 0.05	**0.22** ± **0.05**
50	1.39 ± 0.002	**−0.50** ± **0.069**	−0.41 ± 0.008	−0.65 ± 0.05	0.34 ± 0.15
100	1.370 ± 0.001	−0.511 ± 0.008	−0.439 ± 0.108	−0.450 ± 0.007	0.877 ± 0.022

Note

Wilcoxon signed‐rank test is used to compare observed trait values with the mean values of 999 null models expectation. The mean test was two‐tailed, all other tests were one‐tailed, we report the absolute value of the effect size. Boldface type indicates *p* < .05. SDNN, standard deviation of nearest trait axis neighbor in a quadrat. Specifically, all the values of significance are test whether the observed trait distributions were significantly smaller or less than expected by chance as we predicated, except for the mean test according to a priori assumptions. For example, a significant Kurtosis value indicates a more flat curve of traits distributions than random assemblies, and a significant SDNN value represents species spaced more evenly along trait axes

Lastly, trait and phylogenetic tests tended to identify the same quadrats as possessing nonrandom patterns associated with functional and phylogenetic filtering at the 10 m (*χ* ^2^ = 17.908, *p* < .01) and 20 m (*χ* ^2^ = 3.488, *p* = .05) scales. There was no significant congruence between the quadrats that identified as nonrandom referring to functional and phylogenetic overdispersion (Table 4).

**TABLE 4:** Congruence between phylogenetic and trait‐based tests of community assembly at four nested spatial scales

Scale	Clustered		Overdispersion	
	*χ* ^2^	*p*	*χ* ^2^	*p*
10 m	17.908	**<.01**	0.737	.39
20 m	3.488	**.05**	0.844	.36
50 m	0.005	.95	0.12	.84
100 m	0	1	0	1

Note

Individual quadrats were scored for the presence of phylogenetic test indicating phylogenetic clustering and trait‐based test consistent with habitat filtering. A chi‐square test (*df* = 1) was used to test the hypothesis that trait‐based and phylogenetic‐based tests identified the same quadrats. The process was repeated for phylogenetic tests indicating even dispersion and trait‐based tests indicating even spacing. Boldface type indicates *p* < .05.

## 4 DISCUSSION

### 4.1 Mismatch between phylogenetic relatedness and functional traits

In this study, we quantified the phylogenetic signal of six plant functional traits for the 162 species in a subtropical forest in China. All traits showed significant, but weak, phylogenetic signal (Table 1). This may indicate that the phylogeny could be expected to be a rough proxy for trait similarity at the quadrat scale (Cadotte, Albert, & Walker, 2013; Swenson et al., 2007). However, when we looked at the community structures reflected from the two methods, it showed both similarities and differences between them. On the one hand, we detected phylogenetic and trait clustering (reflected by reduced variances and ranges of functional traits) across the entire FDP at all scales (Table 2 and Table 3). Thus, phylogenetically and functionally similar species tended to co‐occur (Kraft et al., 2008; Swenson et al., 2007). This may be taken as evidence of competitive exclusion of functionally dissimilar species in a given environment or environmental filtering sensu stricto (Kraft, Adler, et al., 2015; Mayfield & Levine, 2010; Webb, 2000). On the other hand, we found no evidence for significant phylogenetic overdispersion at small scales, whereas the trait‐based analyses presented here showed even trait dispersion patterns at small scales which indicated co‐occurring species are more dissimilar from one another than predicted. This suggests that niche differences may be important at small scales in the BDGS FDP (Baraloto et al., 2012; Cavender‐Bares, Ackerly, Baum, & Bazzaz, 2004; Kraft, Cornwell, Webb, & Ackerly, 2007; Kraft et al., 2008).

The present study has shown that in some cases there is congruence in phylogenetic and trait‐based patterns, while in other cases these results are incongruent. Similar results for tree communities have been reported previously. For example, Swenson and Enquist (2009) have reported a mismatch between phylogeny and functional traits in a Neotropical dry forest. In their study, they indicated that the reason may be a lack of phylogenetic signal in functional traits basally and terminally in the phylogenetic tree. Thus, only functional traits with a high degree of basal and terminal phylogenetic signals (maximum height of trees in that study) were consistent with their phylogenetic results. A similar inconsistency was found in the Xishuangbanna tropical forest plot in Yunnan, China (Yang et al., 2014). Thus, when there are multiple potentially opposing mechanisms and little‐to‐no phylogenetic signal in trait data, phylogenetic and functional trait‐based analyses will have varying levels of congruence as seen in the present study. Despite these inconsistencies, phylogenies may still capture additional information that is not contained in the traits that can be measured and may, therefore, still be a useful indicator of non‐random processes (Swenson, 2013, 2019; Swenson et al., 2017; Zambrano et al., 2017).

### 4.2 Scale dependence of the community processes

The means NTI increased at larger spatial scales (Table 2), which indicated that phylogenetic clustering was stronger at larger spatial scales and this general pattern was also found in the trait data. Thus, similar species tend to cluster on large scales, which is indicative of competitive exclusion of dissimilar species via performance differences (e.g., Mayfield & Levine, 2010) or environmental filtering sensu stricto. It is impossible to disentangle these two possibilities without detailed assays of species performance in all environments while removing species interactions, but both possibilities indicate the overall importance of the abiotic environment in driving the structure and assembly of the tree community at large scales. Even trait dispersion was found at the 10 × 10 m, 20 × 20 m, and 50 × 50 m scales, but not at the largest spatial scale (Table 3, Table A1). This overall trend is similar to previous scale dependency work in phylogenetic and trait‐based community ecology where the abiotic environment is more important at large scales and biotic interactions are more important at fine scales (Cavender‐Bares, Keen, & Miles, 2006; Grime, 2006; Kraft et al., 2008; Kooyman, Cornwell, & Westoby, 2010; Kraft & Ackerly, 2010; Qian, Hao, & Zhang, 2014; Swenson & Enquist, 2009; Falster, Brännström, Westoby, & Dieckmann, 2017).

### 4.3 Community processes reflected by functional traits

In this study, we found LT and LNC were clustered at all scales, indicating the important effects of the environmental factors on community structure and assembly. Generally, variation in LT is thought to be linked to plant responses to light (Mendes et al., 2001; Urbas, 2000). Nitrogen is an important nutrient in plants, and most nitrogen is concentrated in chloroplasts, mainly used to form RuBisCo and therefore greatly regulating the Calvin Cycle. Therefore, nitrogen determines the key factor of photosynthetic material metabolism and plant growth in the process of photosynthesis (Hikosaka, 2004; westoby & wright, 2006), which is the key limiting factor of leaf photosynthesis. In general, these patterns of trait dispersion were correlated with topographic and soil variables including elevation, convexity, and soil fertility (Tables A2 and A3) particularly on larger spatial scales. This further underscores the importance of deterministic processes and the abiotic environment in driving tree community assembly in the forest plot. It is important to note, that many abiotic variables co‐vary with topographic gradients including many not measured presently. Additionally, due to working in diverse systems of large long‐lived organisms, experimental manipulations are not feasible. Thus, we are unable to definitively infer the importance of one abiotic variable over another given the data and analyses.

Our analyses of LA and LPC found significant overdispersion and the SLA results found both clustering and overdispersion. In other words, species with dissimilar values for these traits were, typically, more likely to co‐occur in the forest plot. This local scale observation is the pattern expected from niche differences driving community assembly. Thus, we find evidence for multiple deterministic processes driving the structure and assembly of the tree communities in this forest with the abiotic environment being important across spatial scales and the importance of functional differentiation only being detectable on finer spatial scales. Finally, while we find evidence of deterministic processes, we also find patterns of phylogenetic and trait dispersion that are hardly, or not at all, distinguishable from a random pattern. This result is congruent with previous analyses of this forest focusing on beta diversity where there is evidence for both deterministic and stochastic processes (Qiao et al., 2015). Though, whether the random patterns in both of these papers are truly attributable to stochasticity or whether they are due to opposing deterministic processes (e.g., Swenson & Enquist, 2009) is still uncertain. Disentangling these possibilities would require further study including experimentation.

## 5 CONCLUSIONS

The phylogenetic and trait‐based tests we have conducted in this study show that the structure and assembly of the tree community in the 25‐ha BDGS FDP are largely nonrandom. The results show that clustering of similar species occurs across most scales and species with dissimilarity on a few trait axes co‐occur locally. From this, we can infer that the abiotic environment plays a major role in driving species distributions and co‐occurrences either through competitive exclusion of dissimilar species or environmental filtering sensu stricto. Niche differences do also play a role locally, as indicated by patterns of trait overdispersion, and stochasticity is less important, as indicated by the non‐random phylogenetic and trait results throughout the study. In sum, the results generally reject a stochastic model of community assembly in the forest studied and further indicates the importance of abiotic variation in driving species distributions across scales and the importance of biotic interactions locally.

## CONFLICT OF INTEREST

The authors declare that they have no conflict of interest.

## AUTHOR CONTRIBUTIONS

**Jiaxin Zhang:** Formal analysis (lead); investigation (equal); writing–original draft (equal); writing–review and editing (equal). **Nathan Swenson:** Writing–review and editing (equal). **Jianming Liu:** Investigation (equal). **Mengting Liu:** Investigation (equal). **Xiujuan Qiao:** Funding acquisition (equal); writing–original draft (equal); writing–review and editing (equal). **Mingxi Jiang:** Funding acquisition (equal); project administration (equal); writing–original draft (equal); writing–review and editing (equal).

## Data Availability

The data used in this study are available in the Dryad Digital Repository: https://doi.org/10.5061/dryad.f7m0cfxsd.

## Appendix 1

**TABLE A1:** Wilcoxon signed‐rank test of abundance‐weighted null model results at four nested spatial scales

Trait and Scale (m)	Environmental filtering			Niche difference	
	Mean	Range	Variance	Kurtosis	SDNN
LT					
10	0.43 ± 0.01	0.31 ± 0.03	0.27 ± 0.03	0.29 ± 0.03	**−0.17** ± **0.01**
20	0.722 ± 0.048	**0.175** ± **0.032**	0.159 ± 0.043	0.133 ± 0.023	0.389 ± 0.028
50	0.808 ± 0.021	**0.772** ± **0.008**	0.351 ± 0.027	**0.454** ± **0.001**	0.87 ± 0.027
100	1.433 ± 0.09	−0.175 ± 0.011	0.987 ± 0.044	0.716 ± 0.097	0.92 ± 0.032
LA					
10	**0.31** ± **0.03**	**−0.16** ± **0.11**	−0.66 ± 0.03	0.82 ± 0.01	−0.11 ± 0.02
20	0.92 ± 0.061	**−1.19** ± **0.043**	**0.977** ± **0.49**	**1.203** ± **0.003**	**−0.065** ± **0.05**
50	**0.97** ± **0.05**	0.462 ± 0.04	−0.445 ± 0.02	0.79 ± 0.03	−0.032 ± 0.03
100	0.888 ± 0.09	0.780 ± 0.052	1.699 ± 0.077	0.983 ± 0.007	0.031 ± 0.009
SLA					
10	0.33 ± 0.02	0.16 ± 0.02	−0.72 ± 0.01	−0.68 ± 0.05	0.12 ± 0.01
20	0.25 ± 0.013	**−0.128** ± **0.033**	−0.821 ± 0.016	−0.70 ± 0.011	**−0.175** ± **0.067**
50	**0.877** ± **0.012**	0.410 ± 0.041	−1.093 ± 0.043	**−1.28** ± **0.082**	−0.381 ± 0.012
100	0.229 ± 0.182	1.487 ± 0.059	1.132 ± 0.098	1.323 ± 0.004	−0.789 ± 0.043
LCC					
10	0.22 ± 0.01	0.25 ± 0.01	0.43 ± 0.04	0.28 ± 0.03	−0.46 ± 0.05
20	0.667 ± 0.053	**0.122** ± **0.053**	0.413 ± 0.012	0.135 ± 0.001	−0.482 ± 0.005
50	0.413 ± 0.027	−0.952 ± 0.04	0.615 ± 0.041	0.402 ± 0.008	−0.682 ± 0.05
100	0.162 ± 0.033	−1.045 ± 0.002	0.301 ± 0.072	0.145 ± 0.001	0.783 ± 0.083
LNC					
10	0.55 ± 0.03	−0.74 ± 0.02	−0.66 ± 0.1	0.84 ± 0.05	0.32 ± 0.02
20	**0.775** ± **0.011**	−0.336 ± 0.05	**−0.019** ± **0.05**	**−0.021** ± **0.065**	−0.243 ± 0.028
50	0.355 ± 0.018	**−0.964** ± **0.049**	−0.755 ± 0.009	1.144 ± 0.031	0.477 ± 0.072
100	0.705 ± 0.076	−0.862 ± 0.001	−0.904 ± 0.321	−1.114 ± 0.02	0.905 ± 0.035
LPC					
10	0.63 ± 0.02	−0.32 ± 0.03	−0.66 ± 0.05	**−0.25** ± **0.01**	0.11 ± 0.04
20	0.370 ± 0.052	**−0.184** ± **0.024**	**0.133** ± **0.065**	−0.118 ± 0.05	**−0.25** ± **0.05**
50	0.688 ± 0.002	**−0.504** ± **0.003**	−1.232 ± 0.05	−0.571 ± 0.05	0.412 ± 0.003
100	0.215 ± 0.063	**−0.899** ± **0.018**	−0.889 ± 0.098	−0.750 ± 0.037	**0.473** ± **0.05**

Note

The mean test was two‐tailed; all other tests were one‐tailed. Boldface type indicates *p* < .05. Specifically, all the values of significance are test whether the observed trait distributions were significantly smaller or less than expected by chance as we predicated, except for the mean test according to a priori assumptions. For example, a significant Kurtosis value indicates a more flat curve of traits distributions than random assemblies.

**TABLE A2:** Results of bivariate regression analyses between each community structure index and topographic conditions (topography and soil component) at four spatial scales

	Scale	Elevation	Convex	PC1	PC2	PC3	PC4
NTI	10 m	**0.14**	0.11	−0.06	**−0.16**	0.15	−0.04
	20 m	**0.2**	**0.19**	**−0.17**	0.14	−0.05	−0.02
	50 m	0.13	**0.19**	**−0.13**	**−0.17**	**0.21**	0.01
	100 m	−0.03	0.03	**0.11**	**−0.2**	**0.13**	−0.05
NFI	10 m	**0.27**	**0.2**	0.04	**−0.3**	**0.18**	−0.07
	20 m	**0.36**	**0.34**	0.01	**0.18**	0.1	−0.01
	50 m	0.08	**0.15**	0.01	0.02	0.06	**0.21**
	100 m	**0.17**	**−0.34**	**−0.4**	**−0.18**	**−0.18**	**0.28**

Note

Elevation, convex, and the first four principal components of soil fertility represent for quadrat‐level topographic conditions. Significant standardized regression coefficients were shown in boldface type indicating *p* < .05.

Abbreviations: NFI, nearest function index; NTI, nearest taxon index.

**TABLE A3:** Principal component analyses for thirteen variables of soil fertility at 20 × 20 m scale in the 25‐ha BDGS forest dynamics plot

Soil fertility	PCA1	PCA2	PCA3	PCA4
C	−0.59	−0.35	0.22	−0.21
N	0.88	0.35	−0.18	0.11
δ^13^C	0.75	−0.42	−0.39	−0.24
Bulk density	−0.81	−0.24	0.05	−0.36
PH	0.84	−0.42	0.23	0.14
*T*	−0.77	0.5	0.35	0.08
*p*	−.17	.44	.05	.79
C. Density	−0.4	0.5	−0.72	−0.01
SSN	0.63	0.74	0.11	−0.08
SSC	0.5	0.84	−0.02	−0.12
SS δ^13^C	0.52	−0.68	−0.22	0.06
SS δ^15^N	0.57	−0.04	0.8	−0.02
SSP	−0.21	−0.54	−0.1	0.78
Eigenvalue	5.13	3.35	1.65	1.53
% explained	0.39	0.26	0.13	0.12

Note

Entries are component loadings, eigenvalues, and percentage of variation explained by the first four principal components (PCA1, PCA2, PCA3, and PCA4). Soil samples were taken and analyzed for 13 parameters, including total C and N, P, δ^13^C isotope of two soil layers (0–10 cm and 10–30 cm), pH value, bulk density, soil temperature, C density of first soil layer, and δ^15^N isotope of the subsoil layer.
